# Wheelchair Tilt-in-Space and Recline Functions: Influence on Sitting Interface Pressure and Ischial Blood Flow in an Elderly Population

**DOI:** 10.1155/2019/4027976

**Published:** 2019-03-06

**Authors:** Roland Zemp, Joël Rhiner, Stefan Plüss, Reto Togni, Jan A. Plock, William R. Taylor

**Affiliations:** ^1^ETH Zurich, Institute for Biomechanics, 8093 Zurich, Switzerland; ^2^University Hospital Zurich, Division of Plastic Surgery and Hand Surgery, 8091 Zurich, Switzerland

## Abstract

Pressure ulcers (PUs) result from localised injury to the skin and underlying tissue and usually occur over a bony prominence as a result of pressure, often in combination with shear forces. Both pressure magnitude and duration are thought to be key risk factors in the occurrence of PUs, thus exposing wheelchair-bound subjects to high risk of PU development. As a result, wheelchairs that incorporate tilt-in-space and recline functions are routinely prescribed to redistribute pressure away from their ischial tuberosities. The goal of this study was to analyse the role of full-body tilt and recline angles in governing sitting interface pressure and blood circulation parameters in elderly subjects and thereby investigate the efficacy of tilt-in-space wheelchairs for aiding pressure relief activity. Sitting interface pressure and ischial blood flow parameters were examined in 20 healthy elderly subjects while seated in a tilt-in-space and recline wheelchair. Five different angles of seat tilt (5°, 15°, 25°, 35°, and 45°) were assessed in combination with three different angles of backrest recline (5°, 15°, and 30°). The results of the study show that when compared to the upright reference posture, every position (except 15°T/5°R) resulted in a significant decrease in sitting interface pressure. Ischial blood flow also showed significant increases at four different positions (45°T/15°R, 15°T/30°R, 35°T/30°R, and 45°T/30°R) but only at larger tilt-in-space and recline angles. The results therefore suggest that small tilt-in-space and recline angles are indeed able to reduce sitting interface pressures, whereas changes in ischial blood flow only occur at larger angles. In the literature, cell deformation is thought to be dominant over tissue ischemia in the development of tissue necrosis and PUs. Therefore, together with our findings it can be concluded that frequently undertaking small adjustments in tilt-in-space and recline angle might be important for preventing cell deformation and any associated cell necrosis. Larger angles of tilt-in-space and recline seem to support blood flow returning to the tissues, which is likely to play a positive role in healing damaged tissue.

## 1. Introduction

Localised areas of tissue breakdown in skin and/or underlying tissues often occur in the vicinity of bony prominences due to applied pressure and/or shear forces [1]. These so-called pressure ulcers (PUs) are extremely difficult to treat, exposing the patient to extreme suffering, with several month's incapacitation. In addition, treating a level 4 ulcer [2] can easily cost in excess of $120'000 [3], thereby presenting a huge socioeconomic burden. People that are bedridden or wheelchair reliant are generally at high risk of developing PUs [4]. Typical locations for these injuries include the sacrum, the heel of the foot, and the ischial tuberosities.

A survey covering 25 hospitals in five European countries reported PU rates of up to 18.2%, of which the ischial tuberosities (10.0%) were rated the 3^rd^ most prevalent [5]. Fortunately, US healthcare facilities have reported decreasing prevalence rates from 13.5% in 2006 to 9.3% in 2015 due to increased awareness and PU prevention programs [6], but these injuries remain a major issue in all care settings. 

PUs form when mechanical loading on the tissue leads to localised necrosis [2], with additional factors such as age, soft tissue coverage, activity/mobility, soft tissue integrity, moisture, etc., all known to play a role on the subject's susceptibility [7, 8]. Research has clearly demonstrated that both the magnitude and duration of pressure, as well as their interaction, are key factors for the development of PUs [9, 10]. As early as 1958, the development of PUs in dogs was observed after high pressures were applied to the animals' skin for short durations and low pressures for long durations. However, the precise aetiology as well as the relative importance of each factor towards the development of PUs remains unclear. Two main hypotheses for describing the interdependence between mechanical loading and tissue necrosis exist and propose its aetiology as either cell deformation or tissue ischemia [11, 12]: the first suggests that mechanical loading to the skin induces pressure and shear forces on a cellular level, leading to excessive cell deformation and ultimately tissue necrosis [13]. The second proposes that external pressure reduces blood flow and thus the delivery of sufficient oxygen to the tissue, resulting in necrosis [14]. However, while it is clear that tissue ischemia, venous stasis, and/or poor oxygenation all play a role in tissue aggravation, the relatively faster rate of tissue necrosis due to cell deformation indicates that this is plausibly the predominant mechanism underlying PUs [9, 11, 12, 15]. Conversely, unloading the tissue and increasing blood flow and oxygenation are all likely to play positive roles in the healing of injured tissue [16].

The two most commonly used PU prevention strategies include the use of high-quality wheelchair seat cushions to reduce peak pressure magnitudes through improving pressure distribution, as well as increasing the frequency of pressure relief manoeuvres to reduce the duration of continuous pressure loads [9]. Pressure relief manoeuvres, however, such as wheelchair push-ups, lateral leaning, and forward leaning, can only be performed by individuals with sufficient physical strength and coordination. For users who are not able to independently perform such manoeuvres, wheelchairs with incorporated tilt (inclination of complete seat pan and backrest unit) and recline (additional leaning of only the backrest) functions offer the ability to temporarily redistribute weight and reduce sitting pressure [9]. Several wheelchair studies have reported a decrease in sitting interface pressure [17–26] as well as an increase in ischial blood flow [18, 27, 28] when tilted and/or reclined, compared to upright sitting. However, all of these studies were performed in cohorts of spinal cord injured patients with only one exception, where healthy young subjects were analysed [20]. As a result, there is a clear unmet need to understand the relationships between tilt-in-space and recline and both blood flow and interface pressure in physiologically healthy elderly subjects.

Tilting and reclining are responsible for a partial pressure relief and therefore induce a postocclusive reactive hyperaemia, the vital process by which the human body increases blood flow to tissue that has been deprived of oxygen [29]. Hence, the dwell time in the different sitting positions has to be considered, as blood flow parameters may need time to reach steady state. Tilted and reclined wheelchair positions analysed in previous studies that investigated blood flow were maintained for two [18] or five minutes [27, 28]. Moreover, performing a pressure relief for 3 minutes is known to be more effective for enhancing blood flow than a one-minute relief [28]. It would therefore seem reasonable that any study examining blood flow parameters uses a similar time period to ensure a realistic and reliable assessment of parameters in which a steady state is reached.

The goal of this study was therefore to analyse the role of full-body tilt and recline angles in governing sitting interface pressure and blood circulation parameters in elderly subjects and thereby investigate the efficacy of tilt-in-space wheelchairs.

## 2. Materials and Methods

### 2.1. Subjects

In total, 7 males and 13 females with an average age of 79 years (range: 62-92 years), a mean weight of 77 kg (range: 56-106 kg), and an average height of 166 cm (range: 152-182 cm) participated in this study. Subjects were required to be at least 60 years old and were excluded if they were wheelchair-bound, suffered from acute musculoskeletal injury, cognitively impaired, or diagnosed with a blood pressure disorder or peripheral arterial disease. Participants were recruited in collaboration with two nursing homes (Riedhof, Zurich; Artos, Interlaken), where the measurements were conducted. Each subject was provided with a T-shirt and soft, comfortable trousers, without rear pockets or seams in critical locations, in order to standardise clothing and avoid pressure concentrations while seated. The study was approved by the local Ethics Committee (EK 2018-N-26), and all participants provided written informed consent prior to the measurements.

### 2.2. Wheelchair and Seat Cushion

**Figure 1:**
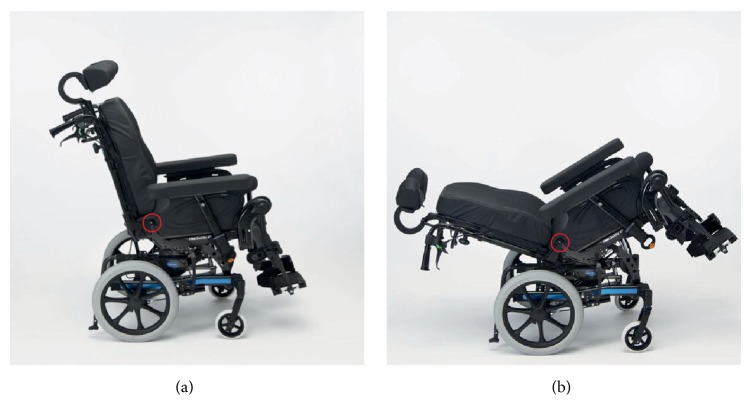
Wheelchair “Rea Dahlia 45” (Invacare®) in the upright reference posture (a) and the maximum tilted/reclined position (b). The pivot point of the backrest is highlighted by a red circle.

The Rea Dahlia 45 wheelchair (Invacare® International GmbH, Witterswil, Switzerland), with standard Flo-shape seat cushion (455x535 mm with Dartex cover) and flex3 backrest, was used for this study (Figure 1). This wheelchair was selected due to its passive tilt-in-space design, which is typically used for geriatric as well as wheelchair reliant individuals with severe physical disabilities. Depending on the height and weight of the participant, either the smaller (390 mm seat width) or larger (440 mm seat width) version of the wheelchair was used. Maximum seat tilt was 45° while the backrest could be reclined up to 30°.

For the purpose of this study the wheelchair was equipped with two spirit levels in order to accurately measure tilt (at the seat frame) and recline (at the backrest frame) angles. Initially, footrest height, seat depth and width, armrest height, and headrest height were all correctly tailored to each subject's anatomy. Special attention was paid to ensure that the hamstring muscles were relaxed, in order to avoid unwanted pelvic movement. Additionally, the tension straps on the backrest were adjusted to suit the individual postural needs of each subject in order to maintain a neutral pelvic position. For some subjects, additional neck pillows were used to facilitate comfortable sitting. The pelvic position of each subject was monitored to ensure that the same sitting posture was maintained throughout all measurements. During the initial adjustment phase, subjects were placed into maximum tilt and recline position to allow familiarisation. Participants who felt uncomfortable were excluded from the study.

### 2.3. Tilt and Recline Angles

Five different seat tilt angles (5°, 15°, 25°, 35°, and 45°T) were combined with three different backrest recline angles (5°, 15°, and 30°R). In order to avoid excessive sitting periods during the measurements (approximately 1 hour), the 15°T/15°R and 35°T/15°R combinations were excluded. Each sitting condition was normalised to the 5°T/5°R upright reference posture, resulting in a total of twelve wheelchair configuration comparisons.

To minimise pelvic movement and subject sliding, the tilt angle was always adjusted prior to the recline angle. The order of the twelve different conditions (tilt and recline) was randomised for each subject. Prior to every test position, subjects were placed into the upright reference posture, hence increasing the number of analysed positions to 24. Previous wheelchair studies [18, 27, 28] and pilot measurements have shown that 2.5 minutes are sufficient to stabilise sitting interface pressure as well as to reach steady state circulation.

### 2.4. Measurement Systems

**Figure 2:**
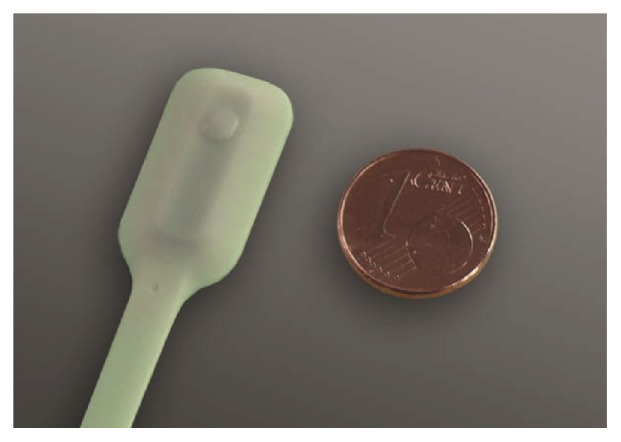
LFx25 probe of O2C (LEA Medizintechnik GmbH).

Sitting interface pressure was recorded using a pressure sensor mat with a 16 x 16 matrix configuration and a size of 392 x 392 mm (Novel Pliance™, Novel GmbH, Munich, Germany), which exhibits an accurate linear relationship with the applied force and low hysteresis [30]. The Oxygen to See (O2C, LEA Medizintechnik GmbH, Giessen, Germany) system was used to record blood flow parameters (Figure 2). The system combines a laser Doppler flowmeter with a tissue spectrometer that allows the capture of blood flow (BF), blood flow velocity (BFV), and relative amounts of haemoglobin (rHb) in arbitrary units (AU).

### 2.5. Data Collection

At the beginning of each measurement, the left ischial tuberosity was palpated while subjects lay on their right side with their hips and knees flexed to 90°. The flat sensor of the O2C system was then attached to the skin covering the tuberosity using adhesive tape (3M Tegaderm™). The pressure sensing mat was fixed over the wheelchair cushion and taped in order to prevent sliding. Once seated, subjects' feet were taped to the footrest in order to help prevent lower limb movement. To ensure a calm and standard temperament a nature documentary was shown throughout all measurements. Both blood circulation and pressure parameters were recorded at a frequency of 1 Hz.

### 2.6. Data Processing

In order to avoid any peaks in pressure due to the O2C system, only pressure values on the right half of the body (8x16 sensors) were considered. In order to reduce background noise and hence improve the signal-to-noise ratio in the BF and rHb signals, the corresponding values were filtered at 0.1 Hz using a 5^th^ order low pass Butterworth filter, according to Sonenblum and Sprigle (2011) [18]. Finally, the last 20 seconds of each (150 second) measurement period was used to calculate the mean sitting pressure [31] (P_mean_; only pressure values greater than 0 kPa were considered) and the average of all blood parameters (BF, V, and rHb). All parameters for the different tilt-in-space and recline positions were normalised to the previous upright reference posture. Data processing was performed using MATLAB (MathWorks®, Natick, MA, USA).

### 2.7. Statistics

A repeated linear mixed model was used to analyse the influence of the test positions (all combinations of tilt and recline) on the mean sitting pressure (P_mean_) and blood parameters (BF, V, and rHb). Subjects were random factors and the covariance type “compound symmetry” for repeated measures was used. A Bonferroni post hoc test was performed to compare the different test positions individually. The significance level was set at p<0.05. All statistical analyses were performed using the IBM SPSS-Software (SPSS AG, Zurich, Switzerland).

## 3. Results

Unfortunately, due to an O2C sensor error, blood flow and blood flow velocity values from 1 subject, as well as relative amounts of haemoglobin of eight subjects, were not captured correctly.

### 3.1. Pressure Parameters

**Figure 3:**
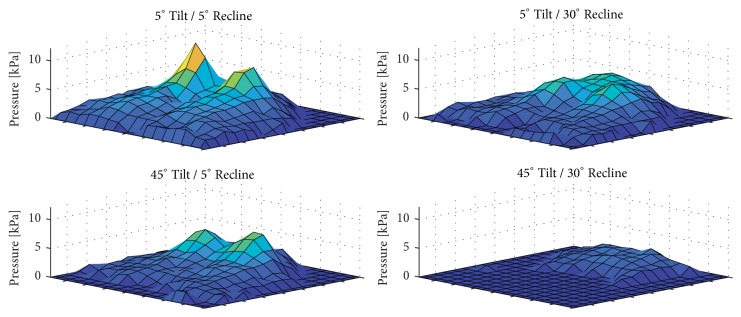
Sitting pressure distribution of an exemplary subject while seated in an upright (5°T/5°R; top left), fully reclined (5°T/30°R; top right), and fully tilted (45°T/5°R; bottom left) as well as fully reclined and fully tilted (45°T/30°R; bottom right) wheelchair position.

**Figure 4:**
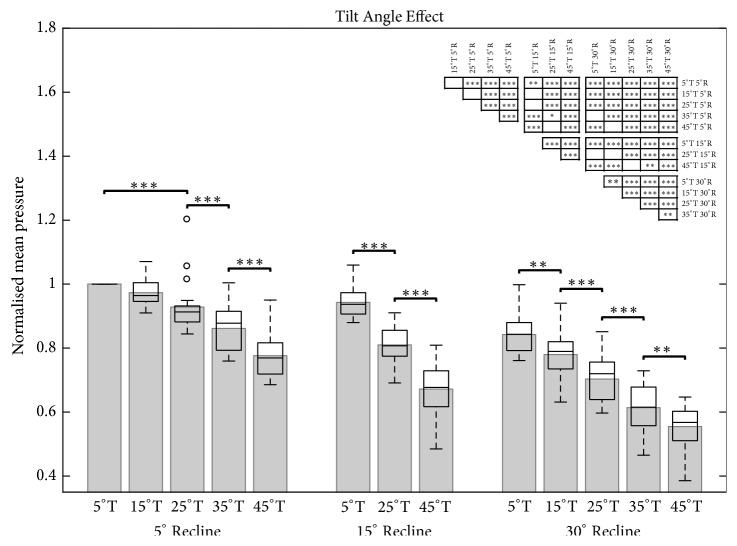
Bar and box plot of the normalised sitting pressure for three recline angles (R; 5°, 15°, and 30°) in response to five tilt angles (T; 5°, 15°, 25°, 35°, and 45°). The first bar on the left represents the upright reference posture (5°T, 5°R). Only neighbouring significance is highlighted in the bar chart. All significant position pairings are illustrated in the significance table (top right). Level of significance (*∗* p<0.05; *∗∗* p<0.01; *∗∗∗* p<0.001).

In general, larger angles of tilt-in-space and recline resulted in lower mean sitting pressures (Figure 3; Supplementary Table S1). In comparison to the upright reference posture, every position except one (15°T/5°R) showed a significant decrease in mean sitting interface pressure (Figure 4). By keeping a 5° reclined position, significant decreases of mean sitting interface pressure could be detected between 25° and 35°, as well as 35° and 45° of tilt. By maintaining a 15° reclined position, significant decreases in mean sitting interface pressure could be detected between all tilt angles (5°, 25°, and 45°). By maintaining a 30° reclined position, significant decreases in mean sitting interface pressure could be detected between all tilt angles (5°, 15°, 25°, 35°, and 45°).

**Figure 5:**
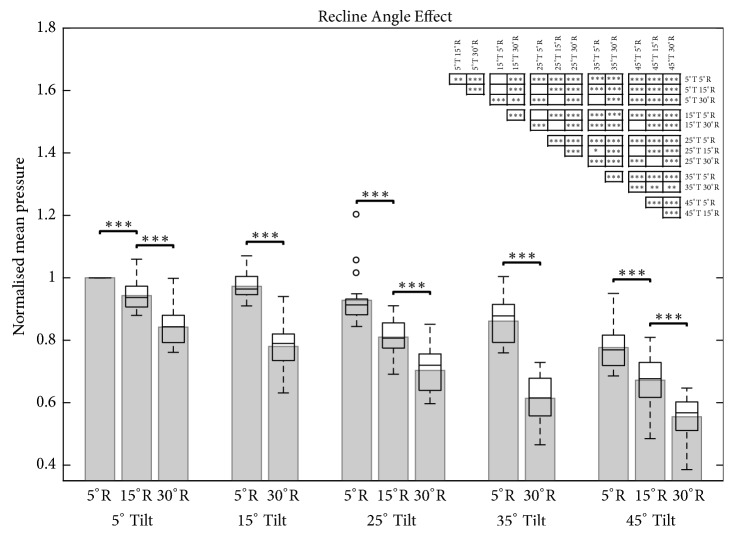
Bar and box plot of the normalised sitting pressure for five tilt angles (T; 5°, 15°, 25°, 35°, and 45°) in response to three recline angles (R; 5°, 15°, and 30°). The first bar on the left represents the upright reference posture (5°T, 5°R). Only neighbouring significance is highlighted in the bar chart. All significant position pairings are illustrated in the significance table (top right). Level of significance (*∗* p<0.05; *∗∗* p<0.01; *∗∗∗* p<0.001).

By keeping the tilt angle constant (5°, 15°, 25°, 35°, and 45°), significant decreases in mean sitting interface pressure could be detected between all analysed recline angles (5°, 15°, and 30°; Figure 5).

### 3.2. Blood Flow

**Figure 6:**
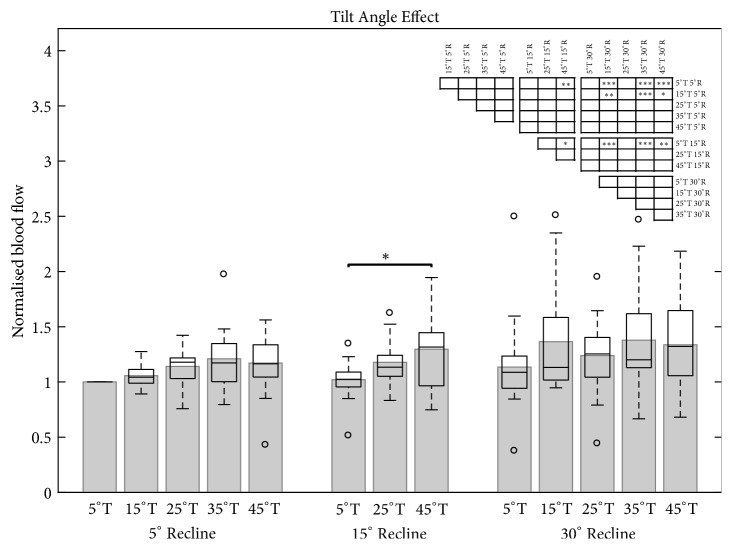
Bar and box plot of the normalised blood flow under the left ischial tuberosity for three recline angles (R; 5°, 15°, and 30°) in response to five tilt angles (T; 5°, 15°, 25°, 35°, and 45°). The first bar on the left represents the upright reference posture (5°T; 5°R). Only neighbouring significance is highlighted. All significant position pairings are illustrated in the significance table (top right). Level of significance (*∗* p<0.05; *∗∗* p<0.01; *∗∗∗* p<0.001).

**Figure 7:**
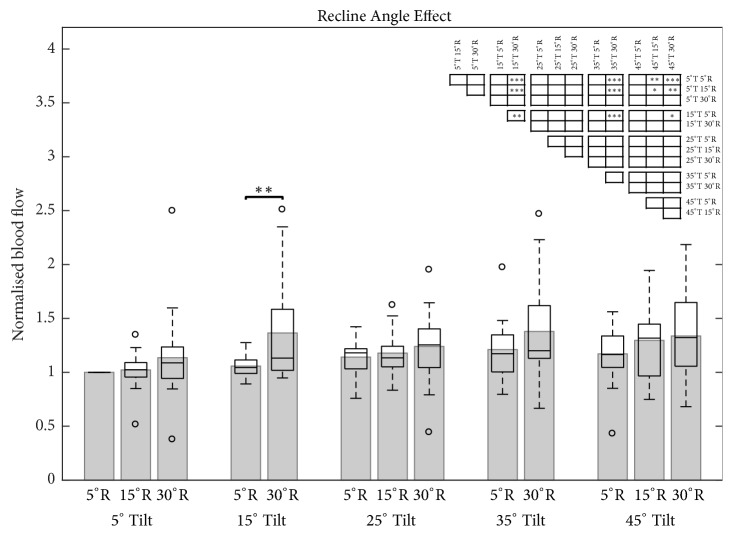
Bar and box plot of normalised blood flow under the left ischial tuberosity for five tilt angles (T; 5°, 15°, 25°, 35°, and 45°) in response to three recline angles (R; 5°, 15°, and 30°). The first bar on the left represents the upright reference posture (5°T; 5°R). Only neighbouring significance is highlighted in the bar chart. All significant position pairings are illustrated in the significance table (top right). Level of significance (*∗* p<0.05; *∗∗* p<0.01; *∗∗∗* p<0.001).

Ischial BF only showed significant increases at large tilt and recline angles (45°T/15°R, 15°T/30°R, 35°T/30°R, 45°T/30°R) compared to the upright reference posture (Figures 6 and 7; Supplementary Table S2).

### 3.3. Relative Amounts of Haemoglobin and Blood Flow Velocity

No significant differences could be found for either rHb or BFV (Supplementary Tables S3 and S4) between any tilt and recline angles.

## 4. Discussion

PUs remain a major challenge for healthcare services globally. Wheelchair users are constrained to prolonged periods of sitting, putting them at increased risk of PU development, thus necessitating effective prevention strategies that enable a reduction of mechanical load at the ischial tuberosities. This study has demonstrated that tilt-in-space and recline wheelchair functions are able to effectively reduce sitting interface pressure and increase ischial blood flow.

### 4.1. Pressure Parameters

From a simple mechanical perspective, the mass of an individual will progressively shift from the seat of the wheelchair to be carried by the backrest as tilt and recline angles increase, resulting in a reduction in sitting interface pressure and possibly also in increased comfort [32]. This study has nicely demonstrated these effects in a practical environment. Since wheelchair users generally spend far less time in a tilt and reclined position compared to an upright position, this increase in backrest interface pressure is rarely relevant for the development of PUs. In the present study, significant decreases in mean sitting pressure compared to the upright reference posture were found for every tilt and recline combination except one (15°T/5°R). This implies that even relatively small increases in tilt and recline angle might be able to result in pressure relief to the tissue. While the consequence of such changes in pressure at the cellular level remains somewhat unknown [33], especially considering thresholds for initiating tissue necrosis [34], the benefits of unloading have been clearly observed in clinical settings [9]. While it is clear that more comprehensive pressure relief activity is always better, the advantage of small compared to large changes in tilt and recline angles is that such activity can be performed easily during the course of a day, possibly helping users to better adhere to more regular pressure relief activities.

While maximal tilt and recline angles of other studies were limited to 35°T and 30°R [17, 22], Aissaoui* et al*. [21] remains the only investigation other than the current in which mean sitting pressure was analysed up to 45°T and 30°R. While young (mean age 21.8 years) rather than elderly subjects were tested in a simulation chair, their study was nicely able to show that significant weight shifts were only observed at >15°T, a point that agrees with our observation of first significant changes in mean sitting pressure. Importantly, however, the present study has demonstrated an additional significant decrease in mean sitting interface pressure between 35° and 45° of tilt when combined with 30° of recline, indicating that wheelchairs that are able to achieve more extreme tilt angles may have an advantage over more conservative versions in terms of pressure relief efficacy.

There is a general consensus in the literature that tilt and reclined positions reduce mean sitting pressure [17–26]. While Stinson* et al*. found only a significant decrease at 30°R (at a constant 5°T) [25], our study already revealed a significant decrease at 15° of recline. Besides the weight shift from the seat to the backrest, pressure reduction by the exclusive use of recline may be explained by a larger contact area created by the coccyx, which was observed during our study. Moreover, as we have already shown in a previous office chair study [31], the material properties and thickness of the sitting cushion (in their case an armchair cushion made from upholstered foam) are likely to play key roles in governing the relative changes in sitting pressure with tilt and recline angles, possibly explaining the small differences in results between these observations.

The results of our study suggest that recline of the backrest alone is able to significantly reduce sitting pressure (Figures 4 and 5), but this sitting posture is known to result in increased shear forces at the body-seat interface. Here, increased surface shear forces of 7% and 25% have been reported for recline angles of 20° and 30°, respectively [23]. Since the damaging effects of shear forces on tissue necrosis are well known [11, 12], the reliance solely on recline functions for wheelchair users is not recommended and rather a combination of tilt and recline is advised. Hobson and coworkers recommended tilting the wheelchair to 25° in order to reduce shear forces to a minimum [23]. Further tilting beyond 25°T, however, is thought to increase shear forces, but in the opposite direction (sliding into the chair), albeit at a reduced pressure force. Although we were not able to measure shear forces within our study, the blood flow parameters (Figure 6) did exhibit interesting behaviour around the 25°T and 30°R position, therefore warranting additional investigation to assess efficacy of this posture for possible pressure relief to the underlying tissue.

### 4.2. Blood Flow

The blood flow parameter outcomes measured in our study were in agreement with the review of Olesen* et al.* [11], who concluded that increasing pressure and shear forces decrease ischial blood flow, suggesting that the indirect use of tilt and recline functions are able to modulate circulation. However, our data indicate that significant increases in blood flow values are only obtainable at large tilt and recline angles (Figures 6 and 7). Here, tilting the seat pan to 45° significantly increased blood flow only when combined with at least 15° recline. Significant increases in blood flow were also found at 15°T, 35°T, and 45°T when combined with 30°R but interestingly, not for 25° tilt. These results support the outcomes of other wheelchair studies that assessed blood flow at different tilt and recline angles, but also the reported significant increases for 25° of tilt combined with 30° of recline [27, 28]. Explanations for this discrepancy could be the high variability in blood flow values across all measurements, possibly skewed by the contrary values of one single subject who exhibited lower blood flow values during tilt and reclined positions than for the upright reference posture. This high variability for all circulation parameters might be explained by the fact that the movement of the musculoskeletal tissue while changing sitting position is extremely individual across subjects, which is consistent with previous observations from an MRI study analysing different sitting positions in office chairs [35, 36].

Considering the increased shear forces [23] and the lack of increased ischial blood flow (our data) during recline alone, it is recommended that reclination is only used in combination with tilt-in-space for minimising the development of PUs. Moreover, no tilt angles showed a significant increase in ischial blood flow at 5° of recline. This fact implies that tilt-in-space functions should always be combined with at least 15° of recline or, even better 30°R, in order to benefit from enhanced blood flow to the ischial tissues.

### 4.3. Practical Relevance

Cell deformation is thought to be dominant over tissue ischemia in the development of tissue necrosis [11]. The results of this study demonstrate that even small amounts of tilt-in-space and recline can be effective in reducing mean sitting interface pressure. Considering the difficulty wheelchairs users have incorporating larger angles of tilt-in-space and recline into their daily lives and the impact this has on daily function, the recommendation to perform small angle changes continually throughout the day is likely to be preferred and more widely accepted by wheelchair users.

Prolonged periods of larger tilt-in-space and recline angles, which showed significant increases in ischial blood flow values, could complement smaller angles of tilt-in-space and recline as blood flow is able to positively affect the healing of injured tissue [16]. However, the time factor remains a controversially discussed topic in the development of PUs. In literature, there is no clear consensus on the duration of time that tilt-in-space and recline should be performed, hence warranting additional investigation into understanding these factors. In addition, there is a lack of knowledge regarding the amount of pressure reduction needed to sufficiently relieve injured tissue, making the development of guidelines on this topic difficult.

One of the main limitations of the current study is the lack of shear force measurements. As shear is well-established as a main causative factor in the development of PUs [11, 12], prevention should focus on reducing shear forces during relief strategies. Hobson* et al.* [23] measured shear forces on the seat interface for two tilt-in-space and recline angles separately, but unfortunately not their combinations. Mechanical loading generates a complex combination of tension, compression, and shear stresses in the tissue. Hence, precise measurement techniques for shear forces inside the tissue at bony prominences do not exist, as information about tissue deformation is highly dependent upon the local material properties and cannot be derived from external shear forces alone [11]. As a result, detailed FE models that are capable of analysing internal shear forces during various tilt and recline angles are clearly needed. Additionally, it must be kept in mind that the probe of the O2C system was directly attached to the skin and that the subjects were sitting on the probe, which could have biased the blood flow parameters due to increased interface pressure. Here, it is important to note that any error in blood flow parameters associated with the O2C system were likely to be greater at more upright sitting postures due to higher sensor pressure differential to the surrounding soft tissues, thus plausibly overestimating the effect of seat-pan tilt. Finally, measurements in this study were performed with healthy elderly subjects, but wheelchair-bound, geriatric, and elderly subjects with severe physical disability may well have different tissue volumes and composition. Changes in tissue composition due to aging or ill health may plausibly influence the thresholds at which sitting interface pressure and blood parameters result in tissue damage, and therefore their influence on PU development.

## 5. Conclusions

The present study has investigated the effects of tilt-in-space and recline angles in modifying mean sitting interface pressure and ischial blood flow in a wheelchair environment. Whereas mean sitting pressure significantly decreased for small tilt-in-space and recline angles, significant increases in ischial blood flow were only observed at larger angles of tilt-in-space and recline. Frequent performance of small adjustments in tilt-in-space and recline angles may therefore be important for preventing cell deformation, which is assumed to be one of the main risk factors in PU development. Larger angles of tilt-in-space and recline were shown to support the return of blood flow back to the tissues, plausibly aiding healing processes in damaged tissue. Blood flow values were highly variable across study subjects, suggesting that changes in sitting posture may result in more subject-specific responses according to, e.g., soft tissue amounts, distribution, and material properties. In order to gain a deeper understanding of the role of forces and tissue properties on cell necrosis and the development of PUs, further investigation is clearly required to determine the role of tilt-in-space and recline angles, as well as their combination on not only sitting interface shear forces, but also the complex 3D loading conditions that occur within the tissue.

## Data Availability

The mean values and standard deviations of all data used to support the findings of this study are included within the supplementary information file.

## Supplementary Materials

Supplementary Materials Table S1: mean value and standard deviation of the normalised mean sitting pressure (P_mean_) for all analysed tilt (T) and recline (R) combinations. Table S2: mean value and standard deviation of the normalised ischial blood flow (BF) for all analysed tilt (T) and recline (R) combinations. Table S3: mean value and standard deviation of the normalised amounts of haemoglobin (rHb) for all analysed tilt (T) and recline (R) combinations. Table S4: mean value and standard deviation of the normalised blood flow velocity (BFV) for all analysed tilt (T) and recline (R) combinations.

## References

[1] Black J., Baharestani M. M., Cuddigan J. (2007). National Pressure Ulcer Advisory Panel's updated pressure ulcer staging system. *Advances in Skin & Wound Care*.

[2] Edsberg L. E., Black J. M., Goldberg M., McNichol L., Moore L., Sieggreen M. (2016). Revised national pressure ulcer advisory panel pressure injury staging system. *Journal of Wound Ostomy & Continence Nursing*.

[3] Brem H., Maggi J., Nierman D. (2010). High cost of stage IV pressure ulcers. *The American Journal of Surgery*.

[4] Grey J. E., Harding K. G., Enoch S. (2006). Pressure ulcers. *British Medical Journal*.

[5] Vanderwee K., Clark M., Dealey C., Gunningberg L., Defloor T. (2007). Pressure ulcer prevalence in Europe: a pilot study. *Journal of Evaluation in Clinical Practice*.

[6] VanGilder C., Lachenbruch C., Algrim-Boyle C., Meyer S. (2017). The international pressure ulcer PrevalenceTM Survey: 2006-2015: a 10-year pressure injury prevalence and demographic trend analysis by care setting. *Journal of Wound Ostomy & Continence Nursing*.

[7] Allman R. M., Goode P. S., Bartolucci A. A., Burst N., Patrick M. M. (1995). Pressure ulcer risk factors among hospitalized patients with activity limitation. *Journal of the American Medical Association*.

[8] Coleman S., Gorecki C., Nelson E. A. (2013). Patient risk factors for pressure ulcer development: systematic review. *International Journal of Nursing Studies*.

[9] Sprigle S., Sonenblum S. (2011). Assessing evidence supporting redistribution of pressure for pressure ulcer prevention: a review. *Journal of Rehabilitation Research & Development*.

[10] Kosiak M. (1959). Etiology and pathology of ischemic ulcers. *Archives of Physical Medicine and Rehabilitation*.

[11] Olesen C. G., de Zee M., Rasmussen J. (2010). Missing links in pressure ulcer research—an interdisciplinary overview. *Journal of Applied Physiology*.

[12] Stekelenburg A., Strijkers G. J., Parusel H., Bader D. L., Nicolay K., Oomens C. W. (2007). Role of ischemia and deformation in the onset of compression-induced deep tissue injury: MRI-based studies in a rat model. *Journal of Applied Physiology*.

[13] Gawlitta D., Li W., Oomens C. W. J., Baaijens F. P. T., Bader D. L., Bouten C. V. C. (2007). The relative contributions of compression and hypoxia to development of muscle tissue damage: an in vitro study. *Annals of Biomedical Engineering*.

[14] Liao F., Burns S., Jan Y. (2013). Skin blood flow dynamics and its role in pressure ulcers. *Journal of Tissue Viability*.

[15] Daniel R. K., Priest D. L., Wheatley D. C. (1981). Etiologic factors in pressure sores: an experimental model. *Archives of Physical Medicine and Rehabilitation*.

[16] Guo S., DiPietro L. A. (2010). Critical review in oral biology & medicine: factors affecting wound healing. *Journal of Dental Research*.

[17] Chen Y., Wang J., Lung C., Yang T. D., Crane B. A., Jan Y. (2014). Effect of tilt and recline on ischial and coccygeal interface pressures in people with spinal cord injury. *American Journal of Physical Medicine & Rehabilitation*.

[18] Sonenblum S. E., Sprigle S. H. (2011). The impact of tilting on blood flow and localized tissue loading. *Journal of Tissue Viability*.

[19] Park U. J., Jang S. H. (2011). The influence of backrest inclination on buttock pressure. *Annals of Rehabilitation Medicine*.

[20] van Geffen P., Reenalda J., Veltink P. H., Koopman B. F. (2008). Effects of sagittal postural adjustments on seat reaction load. *Journal of Biomechanics*.

[21] Aissaoui R., Lacoste M., Dansereau J. (2001). Analysis of sliding and pressure distribution during a repositioning of persons in a simulator chair. *IEEE Transactions on Neural Systems and Rehabilitation Engineering*.

[22] Lung C.-W., Yang T. D., Crane B. A., Elliott J., Dicianno B. E., Jan Y.-K. (2014). Investigation of peak pressure index parameters for people with spinal cord injury using wheelchair tilt-in-space and recline: methodology and preliminary report. *BioMed Research International*.

[23] Hobson D. A. (1992). Comparative effects of posture on pressure and shear at the body-seat interface. *Journal of Rehabilitation Research and Development*.

[24] Sprigle S., Maurer C., Sorenblum S. E. (2010). Load redistribution in variable position wheelchairs in people with spinal cord injury. *The Journal of Spinal Cord Medicine*.

[25] Stinson M. D., Porter-Armstrong A., Eakin P. (2003). Seat-interface pressure: a pilot study of the relationship to gender, body mass index, and seating position. *Archives of Physical Medicine and Rehabilitation*.

[26] Giesbrecht E. M., Ethans K. D., Staley D. (2011). Measuring the effect of incremental angles of wheelchair tilt on interface pressure among individuals with spinal cord injury. *Spinal Cord*.

[27] Jan Y., Crane B. A., Liao F., Woods J. A., Ennis W. J. (2013). Comparison of muscle and skin perfusion over the ischial tuberosities in response to wheelchair tilt-in-space and recline angles in people with spinal cord injury. *Archives of Physical Medicine and Rehabilitation*.

[28] Jan Y.-K., Liao F., Jones M. A., Rice L. A., Tisdell T. (2013). Effect of durations of wheelchair tilt-in-space and recline on skin perfusion over the ischial tuberosity in people with spinal cord injury. *Archives of Physical Medicine and Rehabilitation*.

[29] Bliss M. R. (1998). Hyperaemia.. *Journal of Tissue Viability*.

[30] Hochmann D., Diesing P., Boenick U. (2002). Evaluation of measurement systems for determining therapeutic effectiveness of anti-decubitus ulcer devices. *Biomedical Engineering*.

[31] Zemp R., Taylor W. R., Lorenzetti S. (2016). Seat pan and backrest pressure distribution while sitting in office chairs. *Applied Ergonomics*.

[32] Zemp R., Taylor W. R., Lorenzetti S. (2015). Are pressure measurements effective in the assessment of office chair comfort/discomfort? A review. *Applied Ergonomics*.

[33] Breuls R. G. M., Bouten C. V. C., Oomens C. W. J., Bader D. L., Baaijens F. P. T. (2003). Compression induced cell damage in engineered muscle tissue: an in vitro model to study pressure ulcer aetiology. *Annals of Biomedical Engineering*.

[34] Gefen A., van Nierop B., Bader D. L., Oomens C. W. (2008). Strain-time cell-death threshold for skeletal muscle in a tissue-engineered model system for deep tissue injury. *Journal of Biomechanics*.

[35] Baumgartner D., Zemp R., List R. (2012). The spinal curvature of three different sitting positions analysed in an open MRI scanner. *The Scientific World Journal*.

[36] Zemp R., Taylor W. R., Lorenzetti S. (2013). *In vivo* spinal posture during upright and reclined sitting in an office chair. *BioMed Research International*.

