# Supplementary material for: Wheelchair Tilt-in-Space and Recline Functions: Influence on Sitting Interface Pressure and Ischial Blood Flow in an Elderly Population
Source: Biomed Res Int. 2019 Mar 6;2019:4027976. doi: 10.1155/2019/4027976 (PMC6431370; doi:10.1155/2019/4027976)
Supplement: Supplementary Materials — Table S1: mean value and standard deviation of the normalised mean sitting pressure (Pmean) for all analysed tilt (T) and recline (R) combinations. Table S2: mean value and standard deviation of the normalised ischial blood flow (BF) for all analysed tilt (T) and recline (R) combinations. Table S3: mean value and standard deviation of the normalised amounts of haemoglobin (rHb) for all analysed tilt (T) and recline (R) combinations. Table S4: mean value and standard deviation of the normalised blood flow velocity (BFV) for all analysed tilt (T) and recline (R) combinations. [file 4027976.f1.pdf]

Supplementary Materials

Table S1: Mean value and standard deviation of the normalised mean sitting pressure (P_{mean}) for all analysed tilt (T) and recline (R) combinations.

	5°R	15°R	30°R
5°T	1.000 ± 0.000	0.943 ± 0.046	0.842 ± 0.057
15°T	0.973 ± 0.040		0.780 ± 0.074
25°T	0.928 ± 0.082	0.810 ± 0.060	0.703 ± 0.071
35°T	0.861 ± 0.070		0.614 ± 0.072
45°T	0.776 ± 0.069	0.672 ± 0.080	0.555 ± 0.063

Table S2: Mean value and standard deviation of the normalised ischial blood flow (BF) for all analysed tilt (T) and recline (R) combinations.

	5°R	15°R	30°R
5°T	1.000 ± 0.000	1.021 ± 0.173	1.136 ± 0.411
15°T	1.057 ± 0.096		1.365 ± 0.501
25°T	1.140 ± 0.158	1.178 ± 0.206	1.239 ± 0.329
35°T	1.210 ± 0.272		1.379 ± 0.451
45°T	1.171 ± 0.260	1.296 ± 0.356	1.336 ± 0.418

Table S3: Mean value and standard deviation of the normalised amounts of haemoglobin (rHb) for all analysed tilt (T) and recline (R) combinations.

	5°R	15°R	30°R
5°T	1.000 ± 0.000	0.998 ± 0.040	0.995 ± 0.078
15°T	1.012 ± 0.052		1.009 ± 0.081
25°T	1.015 ± 0.069	0.999 ± 0.085	1.002 ± 0.102
35°T	0.979 ± 0.045		1.003 ± 0.109
45°T	1.003 ± 0.044	0.997 ± 0.086	1.001 ± 0.100

Table S4: Mean value and standard deviation of the normalised blood flow velocity (BFV) for all analysed tilt (T) and recline (R) combinations.

	5°R	15°R	30°R
5°T	1.000 ± 0.000	1.044 ± 0.132	0.978 ± 0.132
15°T	1.007 ± 0.050		1.003 ± 0.143
25°T	0.985 ± 0.098	0.974 ± 0.130	0.975 ± 0.129
35°T	0.983 ± 0.143		0.982 ± 0.148
45°T	0.987 ± 0.135	1.010 ± 0.117	1.022 ± 0.105
